# Precise Estimation of NDVI with a Simple NIR Sensitive RGB Camera and Machine Learning Methods for Corn Plants

**DOI:** 10.3390/s20113208

**Published:** 2020-06-05

**Authors:** Liangju Wang, Yunhong Duan, Libo Zhang, Tanzeel U. Rehman, Dongdong Ma, Jian Jin

**Affiliations:** Department of Agricultural and Biological Engineering, Purdue University, 225 S. University St., West Lafayette, IN 47907, USA; wang3335@purdue.edu (L.W.); duan70@purdue.edu (Y.D.); zhan2693@purdue.edu (L.Z.); trehman@purdue.edu (T.U.R.); ma125@purdue.edu (D.M.)

**Keywords:** RGB camera, filter, low cost, near-infrared, NDVI, machine learning

## Abstract

The normalized difference vegetation index (NDVI) is widely used in remote sensing to monitor plant growth and chlorophyll levels. Usually, a multispectral camera (MSC) or hyperspectral camera (HSC) is required to obtain the near-infrared (NIR) and red bands for calculating NDVI. However, these cameras are expensive, heavy, difficult to geo-reference, and require professional training in imaging and data processing. On the other hand, the RGBN camera (NIR sensitive RGB camera, simply modified from standard RGB cameras by removing the NIR rejection filter) have also been explored to measure NDVI, but the results did not exactly match the NDVI from the MSC or HSC solutions. This study demonstrates an improved NDVI estimation method with an RGBN camera-based imaging system (Ncam) and machine learning algorithms. The Ncam consisted of an RGBN camera, a filter, and a microcontroller with a total cost of only $70 ~ 85. This new NDVI estimation solution was compared with a high-end hyperspectral camera in an experiment with corn plants under different nitrogen and water treatments. The results showed that the Ncam with two-band-pass filter achieved high performance (R2 = 0.96, RMSE = 0.0079) at estimating NDVI with the machine learning model. Additional tests showed that besides NDVI, this low-cost Ncam was also capable of predicting corn plant nitrogen contents precisely. Thus, Ncam is a potential option for MSC and HSC in plant phenotyping projects.

## 1. Introduction

Plant chlorophyll absorbs red light and mostly reflects near-infrared (NIR) light, thus distinguishing plants from other materials such as soil and water [1]. Based on this characteristic, the normalized difference vegetation index (NDVI) is calculated as the ratio between red and NIR reflectance to monitor plant health. Since the 1970s, NDVI has been widely used in remote sensing to discriminate vegetation and other materials in satellite images [2,3,4,5]. It has also been proved that NDVI has significant correlations with green biomass and crop yield [3]. Usually, NDVI is obtained from images captured by a multispectral camera (MSC) or hyperspectral camera (HSC), which captures the accurate red and NIR reflectance of plants [6,7,8,9,10,11,12]. However, both MSC and HSC are heavy, expensive, and require professional operation for imaging and data processing [13,14]. For push-broom hyperspectral cameras in remote sensing, the geo-referencing has also been challenging, whereas the popular multi-lenses multispectral cameras require long imaging distances so they cannot be used in greenhouses and in-door imaging stations. All these issues of MSC and HSC limit their applications in plant phenotyping for the broader agricultural community.

Compared with MSC or HSC, RGB cameras have lower cost, lighter weight, and are easy to operate during imaging. Therefore, RGB cameras are widely used in remote sensing by being mounted to unmanned aerial vehicles (UAV) or ground-based vehicles [15,16]. The standard RGB camera has been explored for calculating a visible NDVI (vNDVI) to estimate NDVI [17,18]. Costa et al. proposed a new vNDVI formula using a genetic algorithm with a standard RGB camera [18]. The vNDVI works as an estimate of NDVI but is obtained with the regular RGB band values instead of the red and near-infrared bands values. 

Another approach for achieving highly precise NDVI is to use an RGBN (NIR-sensitive RGB) camera. A standard RGB camera usually contains a NIR rejection filter, which makes the RGB images close to human vision. After simply removing the filter, an RGBN camera is obtained for capturing the red and NIR reflectance needed for NDVI calculation. Thus, many studies have explored the feasibility of using an RGBN camera to measure NDVI. A two-camera system containing a standard RGB camera and an RGBN camera is reported to calculate NDVI [19]. The standard RGB camera captures the red reflectance, whereas the RGBN camera captures the NIR reflectance. However, the two-camera system creates new challenges, such as image matching and cross-calibration. An RGBN camera with a filter-switching unit is reported to calculate NDVI, getting rid of the limitations of the two-camera system [20]. With the filter-switching unit, the visible image and NIR image are captured by switching the NIR rejection filter, respectively. Subsequently, the NDVI is calculated from both images. However, the position switching of the filter-switching unit is time consuming, which creates the challenge of image matching when the camera is mounted on a moving platform, such as a UAV. An RGBN camera with a new filter to help obtain pure NIR is studied in recent years, which avoids the image matching issue in two-camera and filter-switching systems [1,21]. The new filter is usually either a long-pass filter or a two-band-pass filter. The long-pass filter cuts off the visible light from a particular wavelength, whereas the two-band-pass filter allows two narrow bands of light to pass through. Therefore, the RGBN camera records visible and NIR light in a specified channel(s) depending on the filter applied. Most previous studies calculated NDVI still with the formula (NIR - Red)/(NIR + Red) by using the corresponding channel values obtained from RGBN camera as Red and NIR, respectively [1,16,22,23,24,25,26,27]. However, these results were somehow different from the NDVI by HSC or MSC. Since NIR reflectance is mixed with the visible reflectance in each channel, the pure R and NIR cannot be obtained precisely at the same time with the RGBN camera. Therefore, the NDVI calculated from these earlier reported methods could not be used to replace the NDVI from MSC and HSC completely. 

Targeting this problem, this study proposed an improved NDVI measuring solution based on machine learning and a low-cost imaging system, Ncam. The Ncam was comprised of a widely available RGBN camera, a filter, and a microcontroller. This new NDVI estimation solution was compared with a high-end HSC in an experiment with corn plants under different nitrogen and water treatments in one of the phenotyping greenhouses at Purdue University. Furthermore, additional tests were also conducted on the Ncam images for their potential of assessing other plant physiological features such as nitrogen contents. 

## 2. Materials and Methods

### 2.1. Ncam System Development

**Figure 1:**
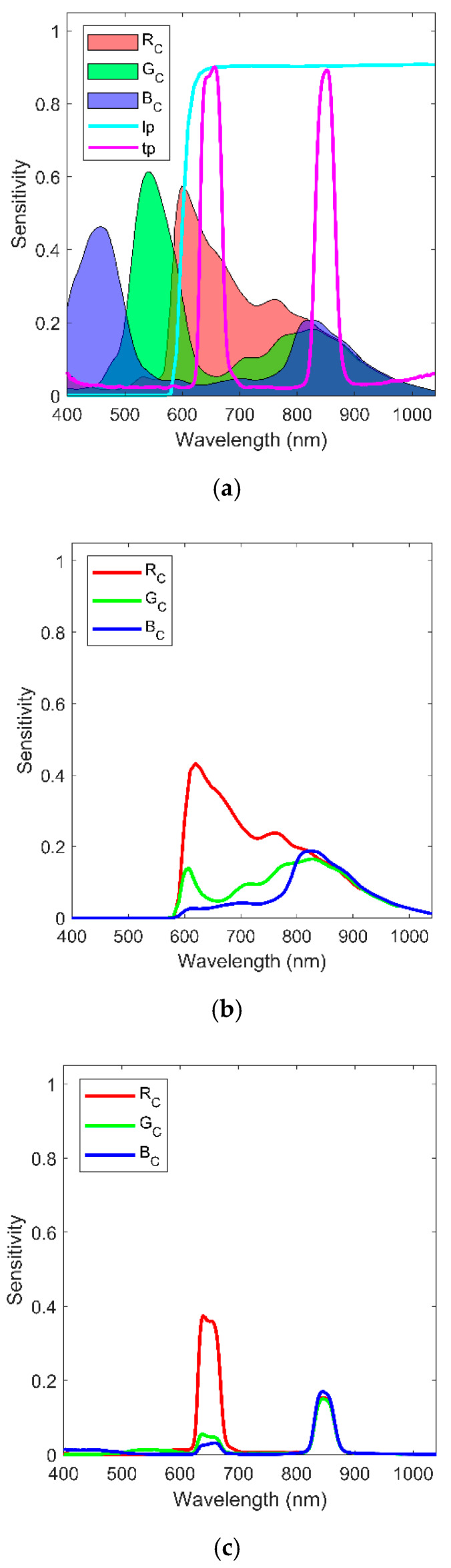
Channel sensitivities of (**a**) Pi camera and long-pass filter, two-band-pass filter, (**b**) Ncam lp, and (**c**) Ncam tp.

Two Ncam systems, Ncam lp and Ncam tp, with different filters, were built for evaluation and comparison. Each of the Ncam imaging systems was composed of one NIR sensitive RGB imaging sensor, a filter, and a microcontroller. The Raspberry Pi Noir camera module V2 (Pi camera, Raspberry Pi foundation, Cambridge, UK) could sense visible and NIR light and was widely available at a low price. Therefore, the Pi camera was selected as the imaging sensor in this study. The value of RGB channels from Pi camera is the product of plant reflectance and each channel sensitivity to different light. It is described with Equation (1).

(1) $$\left[\begin{array}{c} R_{C}\\ G_{C}\\ B_{C} \end{array}\right]=\left[\begin{array}{cccc} a_{1} & b_{1} & c_{1} & d_{1}\\ a_{2} & b_{2} & c_{2} & d_{2}\\ a_{3} & b_{3} & c_{3} & d_{3} \end{array}\right]\left[\begin{array}{c} R\\ G\\ B\\ N\; \end{array}\right]=\left[\begin{array}{cccc} a & b & c & d\; \end{array}\right]\left[\begin{array}{c} R\\ G\\ B\\ N\; \end{array}\right]$$

where *R_C_*, *G_C_*_,_ and *B_C_* are the values of RGB channels, respectively; *R*, *G*, *B*, and *N* are the plant reflectance of red, green, blue, and NIR, respectively; ***a***, ***b***, ***c***, and ***d*** are the sensitivities to red, green, blue and NIR light for RGB channels of Pi camera, respectively. As Figure 1a shows, the RGB channels mostly record the corresponding band reflectance and NIR reflectance; thus, *b_1_*, *c_1_*, *a_2_*, *c_2_*, *a_3_*_,_ and *b_3_* have very small values.

To calculate NDVI, red and NIR reflectance are needed. However, from Equation (1), there are four variables, R, G, B, and N, but only three responses R_C_, G_C,_ and B_C_ are obtained. Red and NIR reflectance cannot be estimated accurately with the original Pi camera. In this study, the filter blocking blue and green light was applied on Pi camera in both Ncam systems to decrease spectral range and increase spectral resolution. In the system of Ncam lp, the blue and green rejection filter (long-pass filter 66043, Edmund Optics, Barrington, New Jersey, USA) cutting off the light before 580 nm was placed in front of the lens of Pi camera. As shown in Figure 1b, each channel of Ncam lp senses red and NIR reflectance from 580 to 1100 nm with different sensitivities. The value of RGB channels for Ncam lp is described with Equation (2).

(2) $$\left[\begin{array}{c} R_{C}\\ G_{C}\\ B_{C} \end{array}\right]=\left[\begin{array}{cc} j_{1} & k_{1}\\ j_{2} & k_{2}\\ j_{3} & k_{3} \end{array}\right]\left[\begin{array}{c} R_{lp}\\ N_{lp} \end{array}\right]=\left[\begin{array}{cc} j & k\; \end{array}\right]\left[\begin{array}{c} R_{lp}\\ N_{lp} \end{array}\right]$$

where *R_lp_* is the red reflectance from 580 nm to 700 nm; *N_lp_* is the NIR reflectance from 700 nm to 1100 nm; ***j*** and ***k*** are the sensitivities to *R_lp_* and *N_lp_* light for RGB channels of Ncam lp; *j_1_* >> *j_2_* > *j_3_* (Figure 1b).

In the system of Ncam tp, a two-band-pass filter (FU-650850LGP, Shenzhen Fuzhe Technology Co., Ltd., Shenzhen, Guangdong, China) transmitting 640 nm (red) and 850 nm (NIR) bands light with full width at half maximum of 40 nm was chosen. As shown in Figure 1c, each channel of Ncam tp senses the narrow red and NIR reflectance with different sensitivities. The value of RGB channels for Ncam tp is described with Equation (3).

(3) $$\left[\begin{array}{c} R_{C}\\ G_{C}\\ B_{C} \end{array}\right]=\left[\begin{array}{cc} l_{1} & m_{1}\\ l_{2} & m_{2}\\ l_{3} & m_{3} \end{array}\right]\left[\begin{array}{c} R_{tp}\\ N_{tp} \end{array}\right]=\left[\begin{array}{cc} l & m\; \end{array}\right]\left[\begin{array}{c} R_{tp}\\ N_{tp} \end{array}\right]$$

where *R_tp_* is the red reflectance from 620 nm to 690 nm, *N_tp_* is the NIR reflectance from 810 nm to 890 nm; ***l*** and ***m*** are the sensitivities to *R_tp_* and *N_tp_* light for RGB channels of Ncam tp; *l_1_* ≫ *l_2_* ≈ *l_3_*, and *m_1_* ≈ *m_2_* ≈ *m_3_* (Figure 1c). 

One of the most popular, low-cost single-board computers, Raspberry Pi 3B (Raspberry Pi foundation, Cambridge, UK), was used as the microcontroller for both systems [28]. Each of them captured raw data and formed an array with a size of 3280 × 2464 and a depth of 16-bit. Then the demosaicing operation was applied in the controller to convert the raw data to an RGB image with a size of 3280 × 2464 × 3 and a depth of 16-bit. The RGB image was finally saved as a Tiff format image to the local SD card.

### 2.2. Plant Samples

A total of 56 corn plants (genotype: Hybrid B73 × Mo17) were grown in the Purdue Lilly Greenhouse (40°25′19.7″ N, 86°55′7.8″ W) for this study. The plants were grown in mixed soil of Greens Grade^TM^ (Profile Products LLC, Buffalo Grove, Illinois, USA) and Metro-Mix^®^ 510 (Sun Gro Horticulture, Agawam, Massachusetts, USA). The temperature in the greenhouse was 23–29 °C, and supplemental lighting was on 12 h from 8 AM to 8 PM a day. To minimize the effects of the microclimate in the greenhouse, all pots (one corn plant per pot) were placed on a closed-loop conveyor system. The system was programmed to run for only 30 s with every 30 min break phase to avoid shaking disturbance to plant growth [29]. Two different water regimes (well-watered and drought-stressed) and two nitrogen levels (low and high) were applied to the plants. The high nitrogen and low nitrogen treatments were achieved with 200 ppm and 25 ppm nitrogen fertilizers, respectively. All plants were irrigated 600 mL water by automatic water station on the conveyor every morning. However, the drought-stressed plants were stopped irrigating four days before imaging. The experiment, consisting of four treatments (two water regimes × two nitrogen levels), was replicated 14 times in a full factorial design.

### 2.3. Image Acquisition

**Figure 2:**
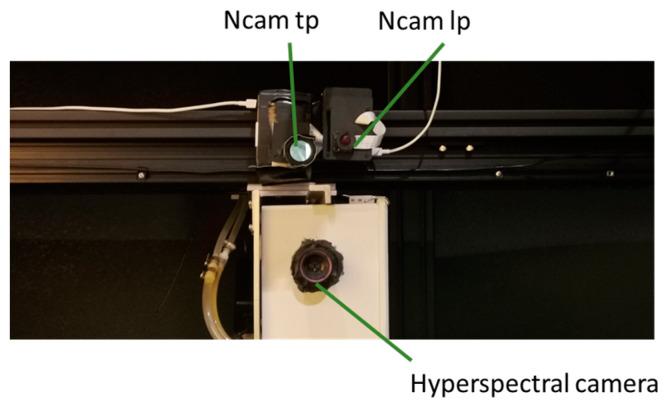
Imaging systems of Ncams and hyperspectral camera.

As the top view image of plants contained more information to monitor plant health than the side view image [30], these two Ncams were mounted on the top gantry in an imaging tower reported by Ma et al. [29]. An HSC (MSV 500, Middleton Spectral Vision, Middleton, WI, USA) was also mounted on the gantry to capture hyperspectral images of plants for NDVI “ground truth” collection (Figure 2). The HSC sensed top view plant reflectance from 380 nm to 1000 nm with a spectral resolution of 1.3 nm. There were eight halogen bulbs above plants inside the tower as the imaging light sources.

Plants were imaged at the V6 stage. The HSC and the two Ncams were initialized before imaging with the parameters shown in Table 1. The corn plants were imaged one by one, and were brought by the conveyor into the imaging tower. To make sure leaves were still while imaging, each plant was imaged 20 s after the conveyor had stopped. The HSC and two Ncams captured images simultaneously to get three images per plant, including two NIR sensitive RGB images and a hyperspectral image. After all plants were imaged, a polyvinyl chloride whiteboard was imaged by these three cameras as white references, respectively. Finally, a total of 171 images (56 plants × three images per plant + three whiteboard images) were duplicated on a computer for further image processing.

### 2.4. Image Processing

**Figure 3:**
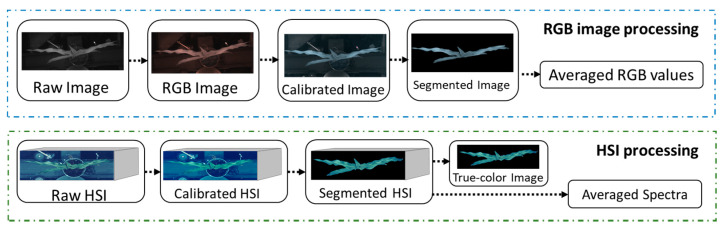
Flowchart of image processing. HSI, hyperspectral image.

The images from Ncams and HSC were processed in Python language (version 3.6.8). The image processing procedure is shown in Figure 3. Firstly, all images of each plant were calibrated when divided by the corresponding whiteboard images, respectively. Secondly, the background was removed by segmentation. For the RGB image from Ncams, *(B_C_ – R_C_) / (B_C_ + R_C_)* was used to segment the image with Equation (4). To segment hyperspectral images of plants, the convolution methodology was applied on the red edge to enlarge the difference between plant and background pixels [30]. Thirdly, all pixel values of the segmented image were averaged, giving the averaged RGB values from Ncams and averaged spectra from HSC for each plant. These averaged RGB values and averaged spectra were written into a table for further data analysis.

(4) $$i_{x,y\;}=\{\begin{array}{c} p_{x,y},n_{x,y}\geq t\\ 0,\;n_{x,y}<t \end{array}$$

where *i_x,y_* is the pixel value of the segmented RGB image, *p_x,y_* is the pixel value of the RGB image after calibration, *n_x,y_* is the pixel value calculated by *(B_C_ – R_C_) / (B_C_ + R_C_)*, and (*x*,*y*) is the index of the pixel in the image.

The true-color image was generated from the calibrated and segmented hyperspectral image to show the actual color of the plants. The RGB values in the true-color image were calculated with a convolution of the pixel value in the hyperspectral image and the sensitivity of a typical RGB camera. The Pi camera with the NIR rejection filter was a typical RGB camera capturing the true-color image, and its sensitivity reported by Pagnutti et al. was used in this study [31]. Then the RGB values of the true-color images were compared with those of Ncams images for channel components analysis.

### 2.5. Data Analysis

#### 2.5.1. Dataset Analysis

Several statistics of the raw dataset, including mean, minimum value, maximum value, skewness, variance, and coefficient of variation, were obtained to analyze the variability of the dataset. The skewness was calculated with Equation (5).

(5) $$Skeness=\frac{\sum_{i=1}^{n}{\left(x_{i}-\mu \right)}^{3}}{ns^{3}}$$

where $x_{i}$ is the value of the i^th^ sample; μ is the mean of the samples; n is the sample size; s is the standard deviation. 

#### 2.5.2. Data Preprocessing

Due to the light source distribution and plants’ 3D structure, the effect of ununiform lighting could not be removed by the calibration based on the flat whiteboard. To reduce the impact, the value of each channel of Ncams was scaled by dividing the sum of its corresponding RGB channels’ values. For the averaged spectra from HSC, log transformation, multiplicative scatter correction, and mean center operations were used to extract the useful signals. As the preprocessed RGB values of Ncams were summed to 1, we did not include preprocessed *G_C_* values to avoid data redundancy in the following modeling.

#### 2.5.3. NDVI Estimation

NDVI from Ncams images was estimated with two types of methods: machine learning algorithms with the values of all channels and the typical formula method with the values of blue and red channels. The results from these two methods were discussed and compared with that from HSC.

The machine learning algorithms, including multiple linear regression (MLR), Gaussian process (GP), and support vector regression (SVR), were applied to the preprocessed *R_C_* and *B_C_* values from Ncams to estimate NDVI. MLR, GP, and SVR are typical regression methods based on different principles and are suitable for different kinds of data. MLR is a widely used linear regression method that is easy to compute and interpret. In contrast, GP and SVR are robust for non-linear data due to the kernel tricks to the models [32,33]. Thus, these three methods were selected for NDVI prediction modeling. When training the model, the MLR models without interaction were fitted, and the kernels with the highest accuracy of GP and SVR models were selected for analysis. The leave-one-out cross-validation was applied to avoid overfitting. Additionally, the NDVI “ground truth” (NDVI_HSC_) for the model development was extracted from the hyperspectral images. The NDVI_HSC_ was calculated with Equation (6).

(6) $$NDVI_{HSC}=\frac{b850-b680}{b850+b680}$$

where *b850* and *b680* are the intensity at 850 nm and 680 nm for each hyperspectral image, respectively.

To evaluate the performance of each model and camera, the R2, RMSE, mean absolute error (MAE) and mean percentage error (MPE) was used as the evaluation metrics. MAE is the mean of the absolute difference between the NDVI from the HSC and the predicted NDVI from Ncams using different models; MPE is MAE divided by the NDVI from the HSC. Besides, the Bland-Altman diagram was used to compare the NDVI measurements from Ncams and the HSC.

The typical formula method for NDVI estimation is used widely with the RGBN camera. It obtains NDVI by replacing the red and NIR reflectance in the calculation formula with the corresponding channel values [21]. In the Ncam system, the red and NIR reflectance were recorded mostly in the red and blue channels, respectively. Therefore, the NDVI from the typical formula method was also calculated with Equation (7) for each plant. Then the calculated NDVI was regressed to NDVI_HSC_. The R2 and RMSE of the regression were obtained for evaluation.

(7) $$NDVI=\frac{B_{c}-R_{c}}{B_{c}+R_{c}}$$

where *B_C_* and *R_C_* are the averaged values of blue and red channels from each Ncam image, respectively.

#### 2.5.4. Other Applications

To explore the additional potential of Ncams on plant health monitoring, the abilities to predict nitrogen contents were discussed briefly in this paper. To train the prediction model of nitrogen contents, the ground truth nitrogen contents of all collared leaves of each plant was measured by a FlashEA 1112 Nitrogen and Carbon Analyzer (Thermo Fisher Scientific, Waltham, MA USA) after imaging.

MLR, which was one of the most common methods for regression, was selected for the prediction of nitrogen contents for Ncams. The performance was evaluated by comparing it with HSC. The partial least square regression was applied to predict the nitrogen contents of plants for HSC. The leave-one-out cross-validation was introduced when training models to avoid overfitting. The R2, RMSE, MAE, and MPE were computed for evaluation. All models in this study were developed with Statistics and Machine Learning, and PLS toolbox in MATLAB (2019a).

## 3. Results and Discussion

### 3.1. Dataset Analysis

The NDVI from HSC ranged from 0.587 to 0.749, with a mean of 0.686, a variance of 0.002, and a coefficient of variation of 0.058. The distribution of NDVI was slightly left-skewed with the skewness of −0.334 but still was approximately normal. 

**Figure 4:**
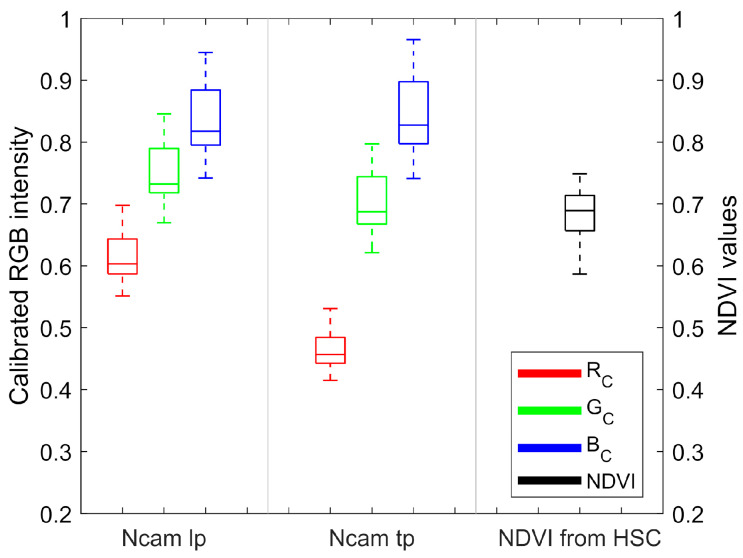
The boxplot of the corn plants raw dataset from Ncams and HSC after calibration with the whiteboard.

**Figure 5:**
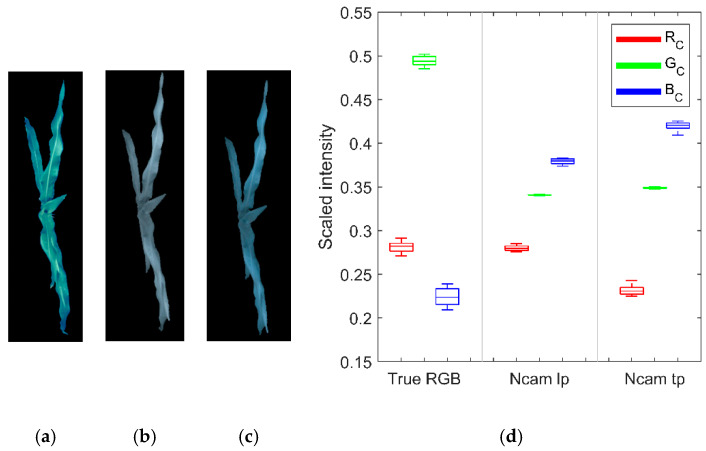
Images of (**a**) true-color RGB, (**b**) Ncam lp, and (**c**) Ncam tp; and (**d**) Preprocessed RGB values of true-color RGB and Ncams images.

The distributions of RGB values of Ncams were all right-skewed, with skewness ranging from 0.420 to 0.533 (Figure 4), which was consistent with the distribution of the plants’ height. As the light source of the imaging tower was above the plant, higher plants tended to be brighter than the shorter plants in the images. Calibration with a flat whiteboard did not remove the light intensity impact on different height plants. Thus, the preprocessing for the dataset by dividing the sum of the corresponding RGB values was necessary. The distributions of the RGB values after preprocessing became more symmetric (Figure 5d).

### 3.2. Channel Components of Ncams Images

The true-color image, which was used to show the actual RGB color of plants, was obtained from the corresponding hyperspectral image. As shown in Figure 5a–c, the true-color image was green due to the plant characteristic reflecting more green light than red and blue. In comparison, Ncams’ images were bluer than the true-color images since the special sensitivities of Ncams. According to Equations (2)–(3), the calibrated value of a channel in Ncams, *C_c_*, could be described with Equation (8).

(8) $$C_{c}=\frac{\alpha N_{p}+\beta R_{p}}{\alpha N_{w}+\beta R_{w}}=\frac{\alpha \lambda N_{w}+\beta \theta R_{w}}{\alpha N_{w}+\beta R_{w}}=\frac{\frac{\alpha }{\beta }\lambda N_{w}+\theta R_{w}}{\frac{\alpha }{\beta }N_{w}+R_{w}}=\lambda +\frac{R_{w}\left(\theta -\lambda \right)}{\frac{\alpha }{\beta }N_{w}+R_{w}}$$

where *N_p_* and *R_p_* are NIR and red band reflectance from the plant, respectively, whereas *N_w_* and *R_w_* are NIR and red band reflectance from the whiteboard; *α* and *β* are the sensitivity of the channel to NIR and red light, respectively; *λ* and *θ* are the reflectance ratio of the plant to the whiteboard in NIR and red bands, respectively; and *α/β* is the sensitivity ratio of NIR to red (NIR-red ratio).

The NDVI value of plants is up to 0.8 [34]. According to Equation (5), plants reflect up to ten times more NIR than red light, which suggests that *λ* is much higher than *θ*. Therefore, the larger NIR-red ratio would give a lager *C_c_* (Equation (8)). In this study, Ncams had the largest NIR-red ratio in the blue channel but the smallest NIR-red ratio in the red channel (Figure 1), thus *B_c_* was the largest whereas *R_c_* was the smallest (Figure 5d). Consequently, the image of Ncams was bluer than the true-color image. Besides, for Ncam, which only sensed red and NIR light in each channel, the NIR-red ratio indicated the purity of red and NIR recorded in the channel. When the NIR-red ratio approximated to zero, the channel would only record red reflectance. Oppositely, when the NIR-red ratio tended to infinite, the channel would only record NIR reflectance. As *C_c_* and NIR-red ratio was positively correlated, it was easy to derive that smaller *C_c_* gave purer red reflectance, and larger *C_c_* gave purer NIR reflectance for the same plant. *B_c_* of Ncam tp was larger than that of Ncam lp, and *R_c_* of Ncam tp was smaller than that of Ncam lp (Figure 5d). Thus, Ncam tp obtained purer red and NIR reflectance than Ncam lp. This finding, while preliminary, suggested that Ncam tp may achieve more accurate NDVI than Ncam lp.

### 3.3. NDVI Estimation

**Figure 6:**
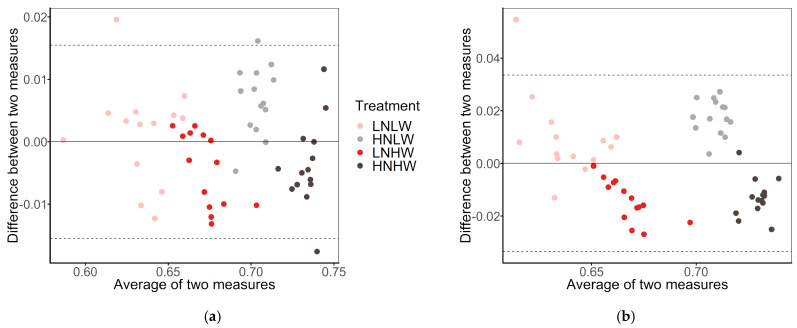
The Bland-Altman diagram of NDVI measurements from (**a**) HSC and Ncam tp, and (**b**) HSC and Ncam lp using the model of MLR. LNLW, low nitrogen and drought-stressed. HNLW, high nitrogen and drought-stressed. LNHW, low nitrogen and well-watered. HNHW, high nitrogen and well-watered.

Ncam tp achieved high performance with both of the models (Table 2). The model of MLR performed a little bit better, with R2 of 0.96, RMSE of 0.0079, MAE of 0.0064, and MPE of 0. 93%, than the other two models. Figure 6a presents the Bland-Altman diagram of NDVI measurements from HSC and Ncam tp using the MLR model. There was no bias between the measurements from Ncam tp and HSC as the points were distributed around zero evenly. The absolute 95% agreement limit was less than 0.02, which indicated a good agreement between these two measurements. 

For Ncam lp, the model of MLR, GP, and SVR achieved close performance. The model of MLR performed a little bit better, with R2 of 0.82, RMSE of 0.0169, MAE of 0.0143, and MPE of 2.09%, than the other two models with respect to MAE and MPE (Table 2). The Bland-Altman diagram of measurements from Ncam lp and HSC in Figure 6b showed that the NDVI for the well-watered plants was underestimated, whereas the NDVI for most drought-stressed plants was overestimated. Regarding the whole dataset, the absolute 95% agreement limit was less than 0.04 and there was no bias, still suggesting a good agreement between these two measurements. 

Ncam tp outperformed Ncam lp with either of the models. One of the possible explanations might be that Ncam tp obtained purer red and NIR reflectance in corresponding channels than Ncam lp, as mentioned in Section 3.2. 

**Figure 7:**
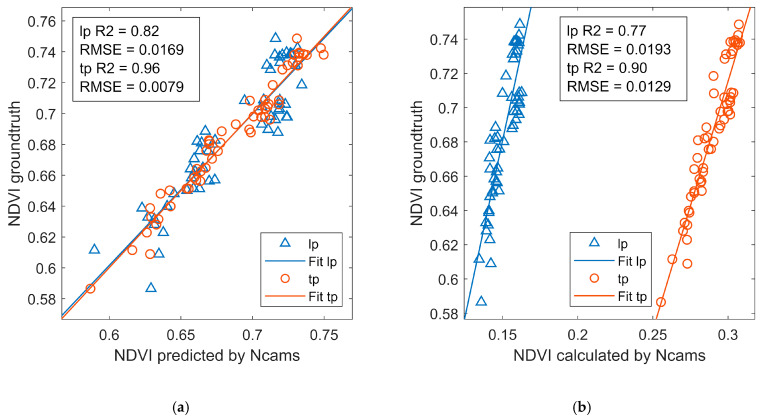
The performances of Ncams on estimating NDVI with (**a**) machine learning methods and (**b**) typical formula. lp, Ncam lp; tp, Ncam tp.

The MLR model was easy to interpret and achieved high performance for both Ncam lp and Ncam tp. Thus, the NDVI estimated by MLR models was compared with that calculated with the typical formula method (Figure 7). The results showed that the MLR model achieved higher accuracy than the typical formula did on NDVI estimation for either of Ncams. The R2 of the MLR model was higher than that of the typical formula by 6% and 7% for Ncam lp and Ncam tp, respectively. The higher performance of MLR models than the typical formula method could be explained from two aspects. First, the NDVI was defined with red and NIR reflectance, which were recorded in both R, G, and B channels in Ncams. The MLR model used all useful information for NDVI estimation by inputting RGB channels values. In contrast, the typical formula method only used blue and red channels’ values. Second, the typical formula method ignored the confounding issue of red and NIR reflectance in the blue and red channels. In the blue channel, Ncams had a large NIR-red ratio, giving an approximate NIR reflectance. However, the red channel record red reflectance as well as NIR reflectance, which was not ignorable. Therefore, the MLR model was more accurate to estimate NDVI compared with the typical formula. 

### 3.4. Other Application

**Figure 8:**
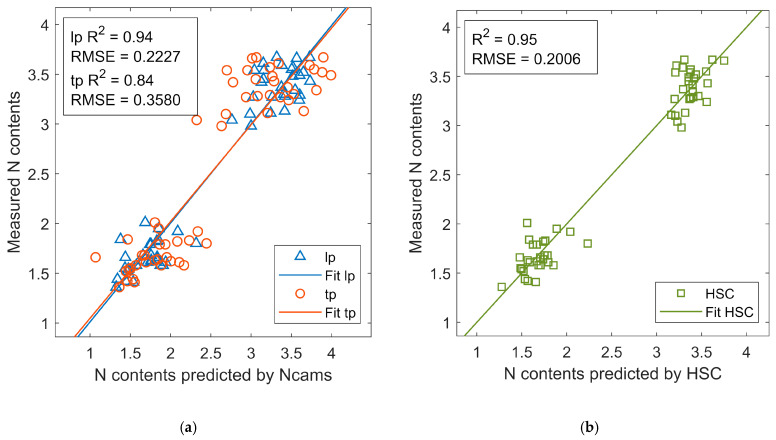
The performances of Ncams on estimating nitrogen (N) contents with (**a**) Ncams and (**b**) HSC. lp, Ncam lp; tp, Ncam tp.

The ability of Ncam to predict nitrogen contents was evaluated by comparing it with the HSC. The MLR model with preprocessed *Rc*, *Bc*, and their interaction was fitted for both Ncam lp and Ncam tp. The partial least square regression model with three latent variables was built for the hyperspectral camera. The results showed Ncam lp achieved a close performance (R2 = 0.94, RMSE = 0.2227, MAE = 0.1737,and MPE = 7.35%) with HSC (R2 = 0.95, RMSE = 0.2006, MAE = 0.1433, MPE = 6.24%), whereas Ncam tp obtained a lower accuracy with R2 = 0.84, RMSE = 0.3508, MAE = 0.2891 and MPE = 11.82% (Figure 8). The reason could be Ncam lp obtained adequate spectrum information for nitrogen prediction, while Ncam tp did not. Researchers comment that nitrogen contents are highly correlated with the visible and NIR reflectance, especially the red edge ranging from 700 nm to 800 nm [35,36]. However, according to Figure 1, Ncam tp did not sense the reflectance at the red edge, which resulted in a low accuracy for nitrogen contents prediction. Therefore, Ncam with the long-pass filter could predict nitrogen contents with high accuracy. 

### 3.5. Ncam Advantages

In the greenhouse, Ncam tp achieved high accuracy in estimating NDVI values of plants, and Ncam lp achieved high accuracy on nitrogen contents prediction for corn plants. Compared to the high-end HSC applied in this study, Ncam had more advantages, such as quicker imaging speed, more compact structure, and easier operation (Table 3). Researchers also proved these advantages of RGB camera compared to HSC [14,37,38]. Moreover, the cost of Ncam is much lower than the HSC. In the Ncam imaging system, the Pi camera costed $24.58, the Raspberry Pi 3 B costed $34.49, the long pass filter costed $25.50, and the two-band-pass filter costed about $10.00. In total, the gross materials cost of Ncam tp and Ncam lp was $69 and $85, respectively. 

### 3.6. Ncam Limitations

The Ncam system was specifically designed for these plant phenotyping researchers who are looking for high-resolution but low-cost sensors for plant NDVI measurement, especially for the indoor phenotyping studies with a light-controlled environment. Many studies have demonstrated that the value of NDVI is easily impacted by the light changes and weather conditions, especially in the field [10,39]. It is unknown how will the weather, sunlight, and the reflection from non-vegetations such as soil and water impact the NDVI measurement with Ncam in the field. Thus, it is necessary to adjust the parameters of Ncam and the machine learning models with field tests to adapt it to the field environment. For nitrogen contents prediction, more nitrogen level treatments and crop species are required to evaluate the performance of Ncams.

### 3.7. Opportunities for Future Work

Compared to the approach getting the NDVI from a standard RGB camera, the NIR sensitive RGB camera is expected to get a higher NDVI estimation accuracy, as NIR reflection is obtained and stored in the RGB bands [1,20]. However, with the development of the application of advanced algorithms, such as genetic algorithm, on the plant phenotyping, a standard RGB camera is proved to be showing a high ability to estimate NDVI values [18]. Thus, it will be valuable to compare the performance to estimate NDVI between a standard RGB camera and a NIR sensitive RGB camera. Based on this, a standard RGB camera accompanying Ncam will be applied in field experiments to predict the NDVI values of plants. The performance of the standard RGB camera and Ncam will be compared to analyze the impact of filters on the estimation of NDVI. Furthermore, we also expected to construct a generalized formula as the substitute of the typical NDVI formula for RGBN cameras based on the model from machine learning. 

## 4. Conclusion

A low-cost solution, Ncam, to estimate NDVI with a widely used RGBN camera was proposed based on machine learning. Two systems with different filters, Ncam lp with long-pass filter and Ncam tp with two-band-pass filter, were developed and evaluated. The Ncams were tested and compared with an HSC in an experiment with corn plants under different nitrogen and water treatments in the greenhouse. The machine learning algorithms, including MLR, GP, and SVR, were applied to estimate NDVI with the ground truth calculated from the hyperspectral image. The results showed: 1) Ncam tp with the machine learning models achieved high performance on NDVI estimation with the R2 of 0.96 and RMSE of 0.0079. 2) The NDVI estimates with machine learning achieved higher accuracy than that calculated from the typical formula. 3) Ncam was also capable of predicting nitrogen contents for corn plants. Therefore, Ncam is an affordable and promising approach to measure NDVI as an alternative to MSC and HSC in the light-controlled environment. In the next experiment, we will test Ncam in the field and evaluate its performance under various weather conditions. 

## Figures and Tables

**Table 1:** Initialization parameters of HSC, Ncam lp, and Ncam tp for the performance testing experiment.

Parameters	HSC	Ncam lp	Ncam tp
Frame rate (FPS)	60	2	2
Exposure time (ms)	6	10	16
Resolution	1760 × 1620	3280 × 2464	3280 × 2464

Note: FPS, frame per second.

**Table 2:** The performances of Ncams on NDVI estimation with different models.

	Ncam tp				Ncam lp			
Model	R2	RMSE	MAE	MPE	R2	RMSE	MAE	MPE
MLR	0.96	0.0079	0.0064	0.93%	0.82	0.0169	0.0143	2.09%
GP Matern	0.96	0.0079	0.0067	0.97%	0.82	0.0168	0.0144	2.10%
SVR quadratic	0.96	0.0081	0.0065	0.94%	0.81	0.0173	0.0149	2.18%

Note: R2, RMSE, MAE, and MPE are from the cross-validated values.

**Table 3:** Comparison of Ncams and HSC.

	Parameters	HSC	Ncam lp	Ncam tp
Configuration	Spatial resolution	1760 × 1620	3280 × 2464	3280 × 2464
	Effective bands	513	3	3
	Spectral range (nm)	400 to 1000	580 to 1000	620 to 690 and 810 to 890
Imaging method	Imaging type	Push broom scan	One-shot	One-shot
	Imaging speed	1 min	Less than 1 s	Less than 1 s
Size and weight	Dimension (mm)	320 × 90 × 96	25 × 24 × 9	25 × 24 × 9
	Weight (kg)	5	About 0.1	About 0.1
Operation	--	Professional	Easy	Easy
Price	--	Over $10,000	$85	$69
